# Mentonian Parasitic Twin: A Rare Case Report

**DOI:** 10.7759/cureus.107741

**Published:** 2026-04-26

**Authors:** Imane Chemlal, Anass Ayyad, Sahar Messaoudi, Mohammed Ech-Chebab, Rim Amrani

**Affiliations:** 1 Neonatology and Neonatal Intensive Care, Mohammed VI University Hospital, Faculty of Medicine and Pharmacy, Mohammed First University, Oujda, MAR

**Keywords:** asymmetric twins, conjoined twins, heteropagus, parasitic twins, siamese twins

## Abstract

Parasitic or asymmetric twins are a rare form of conjoined twins. Thoracopagus and omphalopagus are the most common types, whereas mentonian location remains exceptionally rare. We report the case of a male neonate, born to non-consanguineous parents, who presented with a parasitic twin attached to the left mentonian region, without communication with vital structures, and which was surgically excised. However, the autosite presents a complex congenital heart defect. This case illustrates an extremely rare presentation of parasitic twins located in the mentonian region and highlights the importance of a thorough multidisciplinary evaluation. The prognosis and management depend on the site of attachment, anatomical relationships, and the presence of associated malformations, particularly congenital heart defects, which can be life-threatening.

## Introduction

Conjoined twins are a rare congenital anomaly, with a prevalence of 1.47 per 100,000 births [1]. Asymmetrical conjoined twins, known as heteropagus or parasitic twins, are nonviable and attached to a relatively well-developed autosite. They are much less frequent, accounting for only 3.9% of all conjoined twins, and most commonly present in the thoraco-omphalopagus region [1]. The pathogenesis of this condition remains poorly understood, although prenatal diagnosis is possible through obstetric ultrasound [2].

We report a neonatal case of a mentonian parasitic twin, defined as a parasitic twin attached to the chin, representing an atypical and extremely rare presentation. This location is clinically significant because of its proximity to critical structures, including the upper airway, oral cavity, and cervicofacial neurovascular components, which may complicate surgical and anesthetic management. In addition, the chin region plays a central role in facial aesthetics and identity, making reconstructive outcomes and cosmetic considerations especially important in overall management. This case provides insight into the clinical diagnosis, management, and prognosis of parasitic twins with associated malformations, while highlighting the challenges faced by healthcare professionals in managing this complex condition, as well as its psychological impact on families.

## Case presentation

This patient was a male conjoined twin, born to a 44-year-old grand multiparous mother. The pregnancy was not medically followed. Family history revealed twin births in two paternal uncles. There was no history of consanguinity or hereditary diseases. The mother denied any use of medications or exposure to toxic substances during this unsupervised pregnancy, which was carried to term.

The newborn was delivered at 38 weeks of amenorrhea via vaginal delivery, with a birth weight of 4,100 g. Apgar scores were 7/10 at one minute, 9/10 at five minutes, and 10/10 at 10 minutes, indicating good adaptation to extrauterine life. The newborn was referred to our department on the first day of life for management of a voluminous mass in the left mentonian region.

The mass measured 13 × 8 cm, was painless on palpation, and showed no overlying inflammatory signs. It had a broad-based attachment to the left mentonian region and extended into the oral cavity and lower lip, suggesting significant involvement of the underlying soft tissues. The mass caused functional impairment, including difficulty with oral feeding requiring nasogastric tube feeding, and significant limitation of neck mobility. Despite its size and location, no clinical signs of airway obstruction or respiratory distress were observed.

**Figure 1:**
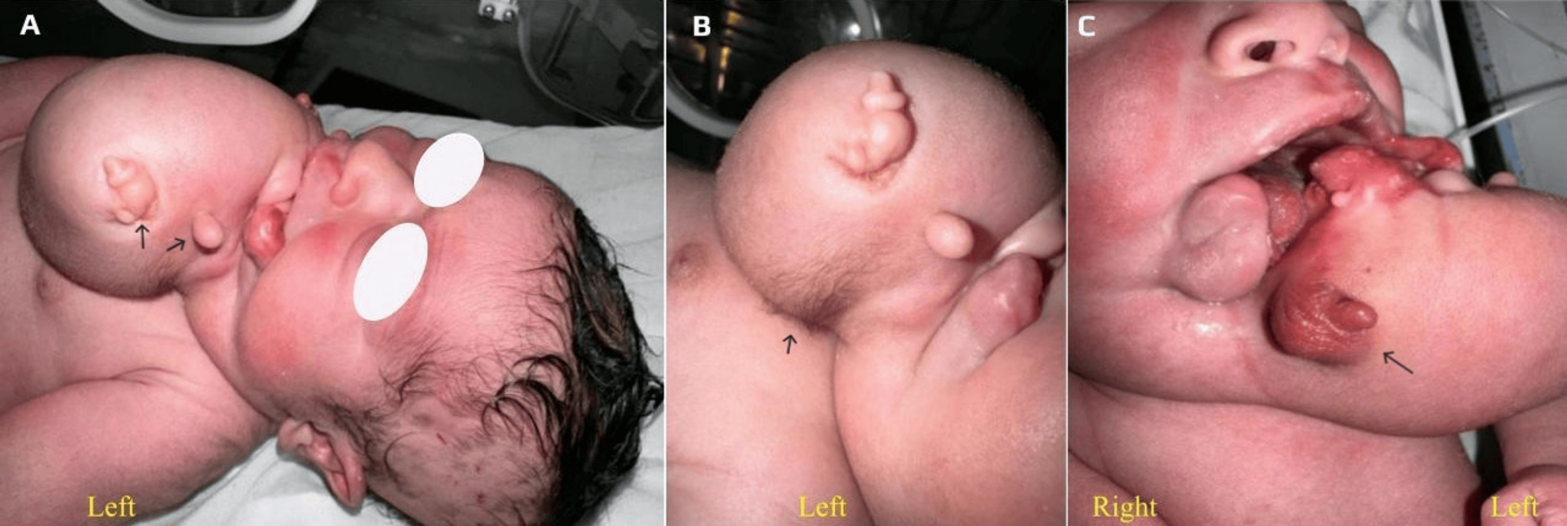
Left anterolateral chin mass with multiple surface structures suggestive of a parasitic twin (A) Presence of rudimentary appendages (arrows) suggestive of limb buds or facial structures (B) Presence of patches of hair (arrow) (C) Presence of external genital structures (arrow) including a scrotum and a penis

On closer inspection, the surface exhibited patches of hair and rudimentary appendages, suggestive of extremities or facial structures (Figures 1A, 1B). Additionally, the genitalia included a well-formed scrotum with a penis measuring 3 mm in length and 2 mm in width (Figure 1C). Oxygen saturation in all four limbs ranged from 89% to 91%, and the remainder of the physical examination was unremarkable

**Figure 2:**
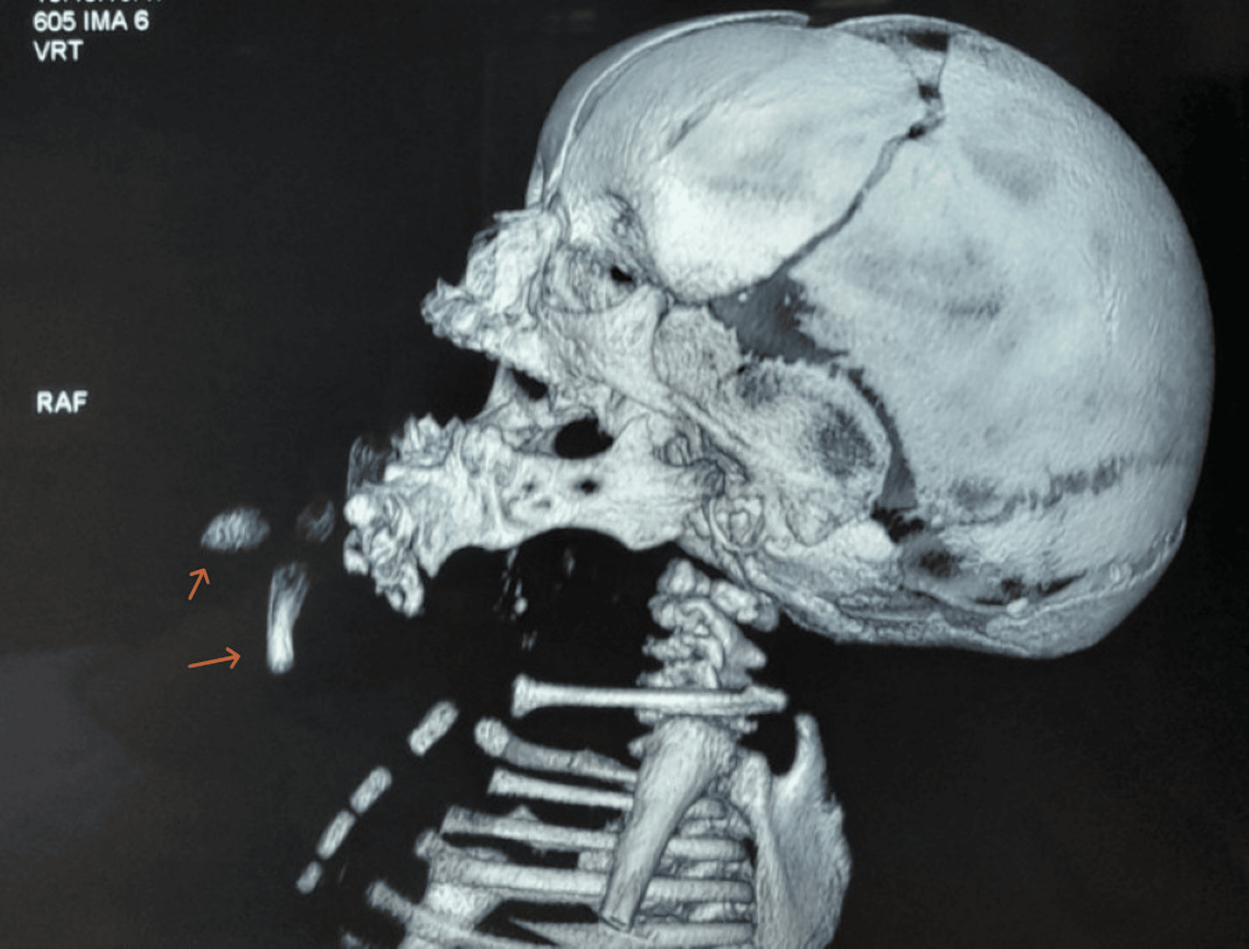
Left lateral view of a cervicofacial CT scan with 3D reconstruction showing osseous structures within a left anterolateral mentonian mass, likely corresponding to long bones of a parasitic twin (arrows), associated with mandibular malformations.

**Figure 3:**
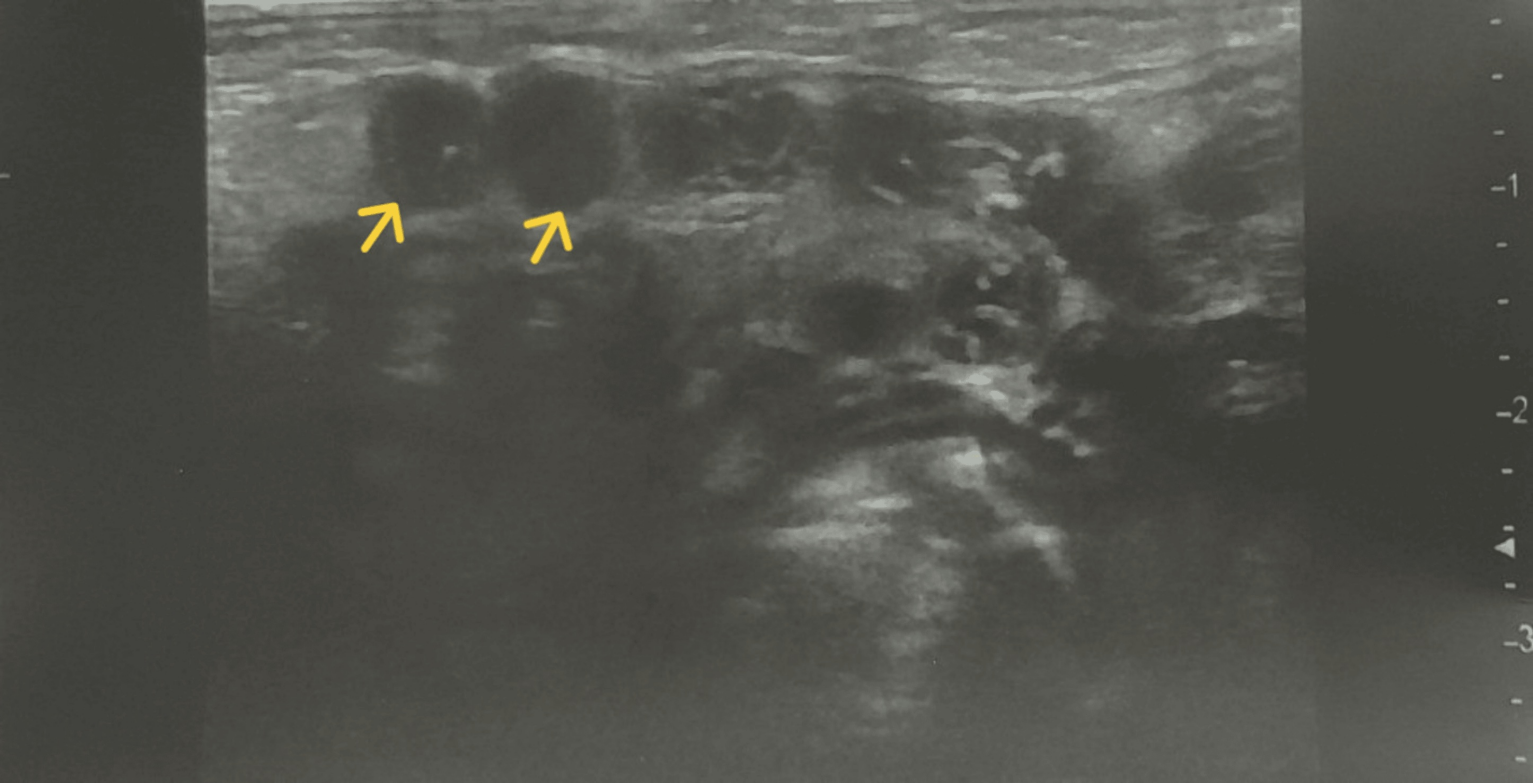
Ultrasound of the mentonian mass showing digestive structures (arrows) consistent with a parasitic twin

Doppler ultrasound, complemented by cervicofacial CT angiography with reconstitution 3D, revealed a left submandibular mass containing osteocartilaginous structures likely corresponding to long bones, soft tissue, fatty structures, and other digestive components, with deformation of the mandible, consistent with a parasitic twin (Figures 2, 3). Screening for associated malformations revealed a complex congenital heart defect on transthoracic echocardiography, consisting of a transposition of the great arteries, a large ventricular septal defect, an atrial septal defect, and pulmonary artery stenosis. The urinary system was normal, and no other malformations were detected.

Laboratory results, including complete blood count and renal and hepatic function tests, were within normal limits. Tumor markers, including alpha-fetoprotein and beta-human chorionic gonadotropin (HCG), were negative.

The clinical and radiological findings strongly supported the diagnosis of a parasitic twin, rather than other differential diagnoses, based on the presence of organized anatomical structures, including external genitalia, limb-like appendages, digestive tract structures, and osseous structures suggestive of long bones.

**Figure 4:**
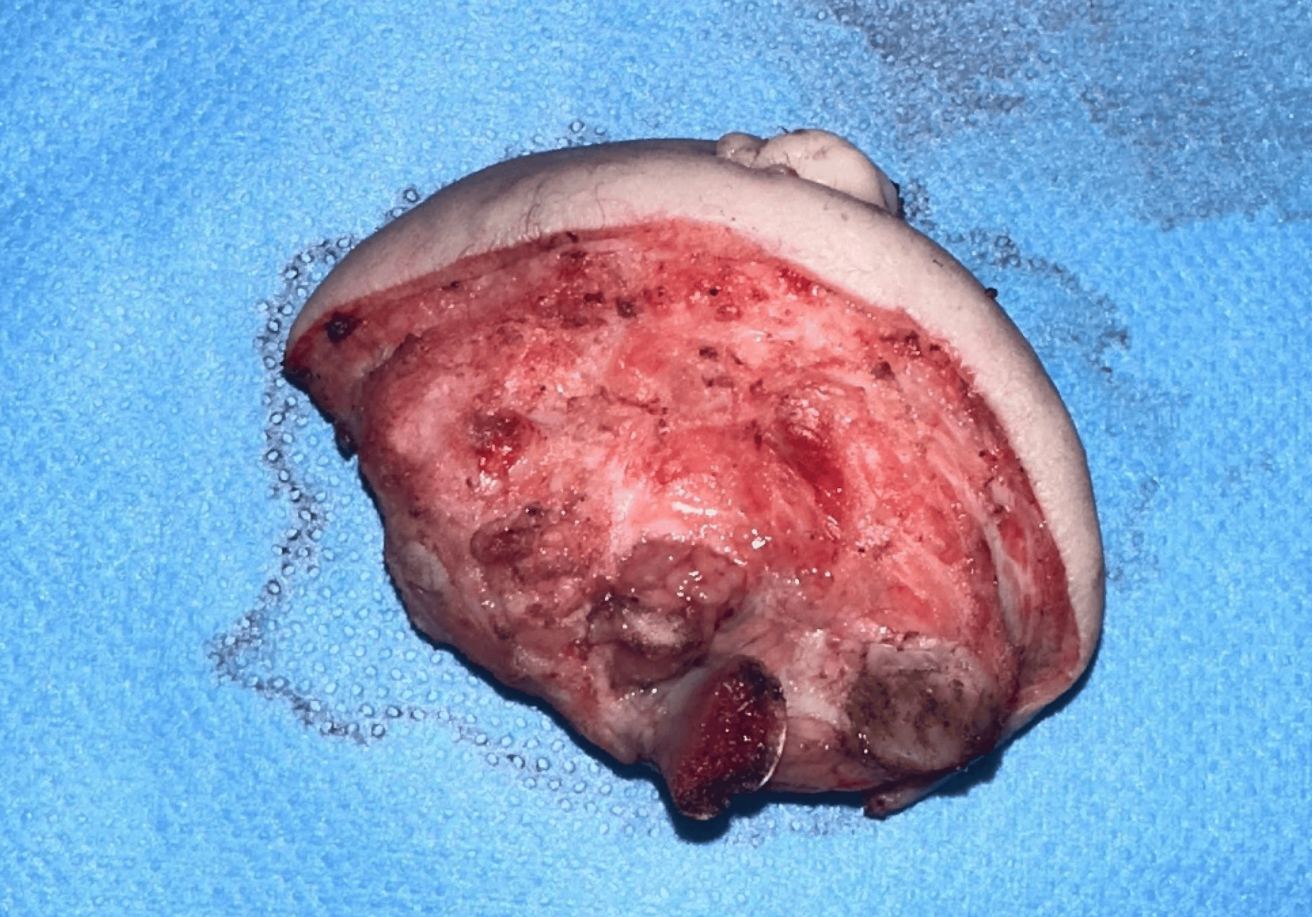
Appearance of the parasitic twin after surgical excision, showing soft tissue and osseous structures

Excision of the parasitic twin (Figure 4) was performed as the initial procedure, with a second-stage plan for plastic and reconstructive surgery. Management required a multidisciplinary team, including neonatologists, pediatric and maxillofacial surgeons, anesthesiologists, and specialized nursing staff. Anesthesia Induction was challenging due to the mass's location, particularly regarding airway management. Intraoperative examination of the parasite revealed no vascular or other communications. The newborn was hospitalized in our neonatal ICU under close surveillance, in anticipation of planned cardiac surgery. However, the patient died before the cardiac intervention could be performed, due to the underlying complex congenital heart disease, which contributed to progressive neonatal hemodynamic instability in a fragile postoperative context. 

## Discussion

Parasitic twins are an extremely rare congenital condition of conjoined twins that predominantly affects males, representing 3.9% of all conjoined twins with an estimated incidence of 1 per 1,000,000 live births [1,2]. It is characterized by an incompletely formed, non-viable parasitic twin attached to its fully formed and viable twin, also called the autosite. It can present as an internal parasite (fetus in fetu) or as an external parasite (exoparasite) [3]. 

This condition represents a significant psychological impact for the mother and the family, with a high stillbirth rate estimated at 40% to 60%, and approximately 35% of affected neonates surviving only one day. The overall survival rate for conjoined twins ranges from 5% to 25% [4]. These outcomes are observed particularly in symmetrical twins, whereas survival is generally better in asymmetric and incomplete forms [2]. 

Early prenatal ultrasound enables the timely detection of this anomaly. Incomplete forms are often diagnosed around 22 weeks of gestation, and sometimes, missed diagnoses may result from suboptimal imaging, late consultations, or misinterpretation [5,6]. In our case, the mother did not receive regular prenatal follow-up, which had a psychological impact at the birth of her baby with this malformation. These conditions compromise preparation for delivery and increase risks for both the mother and the newborn [6].

Multiple classifications are proposed based on the site of fusion, symmetry, and the extent of shared organs. Thoracopagus is the most common type (42%), whereas heteropagus is very rare [1]. Within this spectrum, the present case is notable for its chin location, which poses specific therapeutic challenges because of its proximity to critical structures and potential functional and aesthetic implications. Clinically, the mass showed a heterogeneous yet organized set of structures, including genital structures, rudimentary appendages suggestive of limb buds or facial structures, and hairs. These findings reflect different degrees of anatomical differentiation. Similar variability has been reported in the literature, ranging from intermediate differentiation in upper lip parasitic twins with pseudo-skeletal elements [7], to sacral forms with well-differentiated genital structures [8], and to more advanced organogenesis in epigastric or omphalopagus heteropagus twins showing limb buds, facial structures, and external genitalia [9,10]. Collectively, these observations, including our case, illustrate the broad phenotypic spectrum of parasitic twins.

The main differential diagnosis in this case was a cervicofacial teratoma, particularly an epignathus [11]. Unlike teratomas, which consist of disorganized tissue mixtures without anatomical architecture, the presence of organized structures, such as limb buds and axial organization, supports a diagnosis of parasitic twin rather than a neoplastic process [11]. In our case, both radiologic and intraoperative findings confirmed a structured embryologic organization, reinforcing this diagnosis.

Conjoined twins may present with congenital anomalies classified as thoracic, gastrointestinal, genitourinary, or musculoskeletal [12]. In the present case, a complex cardiac malformation was identified, underscoring the crucial role of echocardiography in early detection of such anomalies. Cardiac malformations are most frequently reported in omphalopagus twins, with an occurrence rate of 39% in autosites compared to 25% in previous series, and ventricular septal defect presents the predominant cardiac anomaly [2]. In our case, a 6 mm ventricular septal defect was identified, consistent with a large defect. They may also share vital organs such as the heart, brain, and liver, and in some cases may have urinary tract communication or intertwined intestines [2]. 

Parasitic twins require a multidisciplinary approach to successful surgical separation, which depends on the site of attachment, anatomical relationships, and associated malformations [2,8]. In particular, when congenital cardiac anomalies are present, a staged approach is generally recommended [13]. Within this framework, the literature supports excision of the parasitic component as the first step of management, followed by delayed correction of associated anomalies [13]. This strategy has been illustrated by Ozkan-Ulu et al., who performed excision of the parasitic twin on day three of life, followed by staged cardiac interventions at 6 and 12 months for complex congenital heart disease [13]. Notably, their case involved severe malformations requiring multiple reconstructive procedures over time, reflecting a high cardiac surgical burden. Our management followed this established principle, beginning with excision of the parasitic twin to relieve functional impairment, optimize feeding and growth, and prevent progressive airway compromise related to the enlarging mass. Delayed cardiac surgery is planned for a later stage, once the patient achieves satisfactory weight gain and has fully recovered from the immediate postoperative period. 

Surgical planning can be optimized using preoperative imaging and prenatal simulation tools, which have further improved outcomes by enabling patient-specific preparation without putting actual patients at risk [14,15]. The procedure becomes highly complex and risky when vital organs, especially the heart and brain, as well as viscera and bones, are shared, or when the autosite presents complex malformations [2,3]. This surgery may need sacrificing one twin for the benefit of the other, an approach that remains controversial [16]. Postoperative mortality of autosites has been reported at 31%, often associated to cardiorespiratory complications and congenital heart defects, as well as surgical stress and challenges in managing physical abnormalities or infections [2,12]. Outcomes are generally more favorable in planned procedures than in emergency surgeries, as thorough preoperative assessment enables a better understanding of anatomy and reduces intraoperative uncertainty [12].

In the present case, intraoperative findings confirmed the radiological assessments, demonstrating well-formed osseous and soft tissue structures without vascular, bony, or visceral connections to the host. Despite successful excision, prognosis remains largely determined by the associated complex congenital heart disease. Although a staged cardiac intervention was planned after recovery from the initial surgery and adequate weight gain, hemodynamic instability related to the cardiac malformation remains a major determinant of outcome. This underscores that, even after technically successful excision of a parasitic twin, severe congenital heart disease can critically affect survival in these fragile neonatal patients.

## Conclusions

Parasitic twins represent one of the rarest congenital malformations and continue to pose significant challenges in neonatal management, requiring a multidisciplinary approach. Prenatal diagnosis is essential to improve care and guide management. Neonatal morbidity and mortality depend on multiple factors, including the presence of life-threatening anomalies, particularly complex congenital heart defects, as observed in our case, which are associated with a poor prognosis. Furthermore, while various anatomical locations of parasitic twins have been described, the mentonian location is exceptionally rare. 
